# The reporting of funding in health policy and systems research: a cross-sectional study

**DOI:** 10.1186/s12961-018-0356-3

**Published:** 2018-08-17

**Authors:** Assem M. Khamis, Lama Bou-Karroum, Maram B. Hakoum, Mounir Al-Gibbawi, Joseph R. Habib, Fadi El-Jardali, Elie A. Akl

**Affiliations:** 1 Clinical Research Institute, American University of Beirut Medical Center, Beirut, Lebanon; 2 Center for Systematic Reviews for Health Policy and Systems Research, American University of Beirut, Beirut, Lebanon; 3 Department of Family Medicine, Faculty of Medicine, American University of Beirut, Beirut, Lebanon; 4 Faculty of Medicine, American University of Beirut, Beirut, Lebanon; 5 Faculty of Health Sciences, American University of Beirut, Beirut, Lebanon; 6 Department of Health Research Methods, Evidence, and Impact, McMaster University, Hamilton, ON Canada; 7 Department of Internal Medicine, American University of Beirut Medical Center, P.O. Box: 11-0236, Riad-El-Solh Beirut, 1107 2020 Lebanon

**Keywords:** Funding, Systematic review, Health policy, Health systems

## Abstract

**Background:**

Major research-reporting statements, such as PRISMA and CONSORT, require authors to provide information about funding. The objectives of this study were (1) to assess the reporting of funding in health policy and systems research (HPSR) papers and (2) to assess the funding reporting policies of journals publishing on HPSR.

**Methods:**

We conducted two cross-sectional surveys for papers published in 2016 addressing HPSR (both primary studies and systematic reviews) and for journals publishing on HPSR (both journals under the ‘Health Policy and Services’ (HPS) category in the Web of Science, and non-HPS journals that published on HPSR). Teams of two reviewers selected studies and abstracted data in duplicate and independently. We conducted descriptive analyses and a regression analysis to investigate the association between reporting of funding by papers and the journal’s characteristics.

**Results:**

We included 400 studies (200 systematic reviews and 200 primary studies) that were published in 198 journals. Approximately one-third (31%) of HPSR papers did not report on funding. Of those that did, only 11% reported on the role of funders (15% of systematic reviews and 7% of primary studies). Of the 198 journals publishing on HPSR, 89% required reporting of the source of funding. Of those that did, about one-third (34%) required reporting of the role of funders. Journals classified under the HPS category (*n* = 72) were less likely than non-HPS journals that published HPSR studies (*n* = 142) to require information on the role of funders (15% vs. 32%). We did not find any of the journals’ characteristics to be associated with the reporting of funding by papers.

**Conclusions:**

Despite the majority of journals publishing on HPSR requiring the reporting of funding, approximately one-third of HPSR papers did not report on the funding source. Moreover, few journals publishing on HPSR required the reporting of the role of funders, and few HPSR papers reported on that role.

## Background

There is growing evidence that source of funding is associated with the reporting of research results. A recent Cochrane methods systematic review found that industry-sponsored studies were more likely than non-industry-sponsored studies to report favourable efficacy results and favourable conclusions (relative incidence increased by 27% and 34%, respectively) [1]. Further, a literature survey of randomised controlled studies published in 2011 found that the majority of studies sponsored by industry reported favourable results [2]. Therefore, the reporting of funding in published evidence might help in interpreting results and highlight possible bias [3]. One study found that physicians were less confident in the results of trials that disclosed industry funding compared to those that did not [4]. In addition, physicians’ willingness to prescribe was higher for drugs assessed in government-funded trials compared to those assessed in industry-funded trials [4]. Another study evaluating 106 review articles found that affiliation of the review author with the tobacco industry was the only factor associated with concluding that passive smoking is not harmful [5].

Guidelines for reporting health research require the reporting of the source of funding and the role of funders given their influence on the design, conduct, analysis and reporting of research [6–8]. International Committee of Medical Journals Editors highlighted the importance of the reporting of funding sources as it could bias the viewpoint and the choice of topics [6]. The latest version of the CONSORT statement (short for Consolidated Standards of Reporting Trials) requires the reporting of the sources of funding and the level of involvement of funders [7]. Similarly, the PRISMA (Preferred Reporting Items for Systematic Reviews and Meta-Analyses) statement underlined the importance of transparent funding reporting as systematic reviews have a critical role in decision-making [8].

While studies have investigated the reporting of funding by papers published in clinical journals [9–12] and the policies of public health journals regarding the reporting of funding, we are not aware of similar studies in health policy and systems research (HPSR). Therefore, the objectives of this study were (1) to assess the reporting of funding in reporting HPSR papers and (2) to assess the funding reporting policies of journals publishing on HPSR.

## Methods

### Overall design

We conducted two cross-sectional surveys, one for papers addressing HPSR and one for journals publishing on HPSR. Below, we describe the details of the two surveys. For both surveys, we considered the following definitions:

- **Funding:** any sort of support to the planning, conduct or reporting of a study, in either monetary or non-monetary form (e.g. logistical support or writing assistance)
- **Funding statement:** any text in the paper that provides information about the funding of the study, including a report of ‘no funding’
- **Funding policy:** a policy that requires, at a minimum, the authors to disclose any source funding of the study

### Survey of HPSR papers

#### Eligibility criteria and search

We included both primary studies and systematic reviews and excluded policy briefs, overviews of systematic reviews, economic evaluation and costing studies, technical reports, conference reports, proceedings, abstracts, editorials and opinion pieces.

To identify systematic reviews, we searched the Health Systems Evidence database of McMaster Health Forum for documents published in 2015 [13, 14]. The Health Systems Evidence database is a regularly updated database of systematic reviews in health policy and systems that relies on different sources of systematic reviews, has its own eligibility criteria, and has an independent and duplicate selection process.

To identify primary studies, we considered 72 journals listed under the ‘Health Policy and Services’ (HPS) category in Web of Science as of June 2016 [15]. We searched for primary studies (e.g. randomised controlled studies, cohort studies, qualitative studies) published in those journals in English in 2016. We considered those studies as eligible if they met the criteria of health systems topics developed by the McMaster Health Forum [13, 14], including governance, financial and delivery arrangements, and implementation strategies.

#### Study selection and data extraction

We developed a data extraction form with detailed instructions using the Research Electronic Data Capture (REDCap) tool hosted at the American University of Beirut. Teams of two reviewers selected studies and abstracted data in duplicate and independently, and resolved disagreements through discussion or with the help of a third reviewer, if needed.

A funding statement could refer to more than one funding contribution. For each study, we collected data on the number of authors, affiliation(s) of the first authors (private or public academic institution, government, not-for-profit organisation, private for-profit organisation, intergovernmental organisation), country of affiliation of the first author and its classification according to the World Bank list of economies issued in July 2015, reporting of study funding (not reported, reported as funded, reported as not funded), reporting of the source of funding (internal fund, governmental, private for-profit and private not-for-profit), and reporting of the role of funders (not reported, reported as ‘no involvement’, reported as involvement in specific stage(s) of research).

### Survey of journals publishing HPSR papers

#### Journal selection

We considered two groups of journals. The first group consisted of all journals listed under the category of HPS by Web of Science as of June 2016. We considered only journals that had an online submission system to review their policies on websites and during the submission process [15]. That resulted in the exclusion of one inactive journal and one active journal that published by invitation only and had no information on reporting of funding on their website. The second group consisted of journals that published the systematic reviews on HPSR included in the first survey.

#### Data extraction

We collected the needed information from the instructions and forms accessible on the journal or publisher’s websites. Teams of two reviewers abstracted data in duplicate and independently using the REDCap tool. They resolved disagreements through discussion or with the help of a third reviewer if needed.

For each journal, we extracted information about whether the journal is categorised by the Web of Science as HPSR, impact factor, International Committee of Medical Journals Editors membership, COPE (Committee on Publication Ethics) membership, affiliation with a professional organisation, requirement of disclosure of conflicts of interest, requirement of disclosure of source of funding, and requirement of disclosure of role of funders.

### Statistical analysis

We conducted descriptive analyses for all variables collected for the included papers and journals. We provided frequencies and percentages for categorical variables, and median and interquartile range for continuous variables. We compared the general characteristics of primary studies with those of the systematic reviews. In addition, we compared journals in which HPSR papers were published with journals listed under the category HPS according to the Web of Science 2016. We used the Mann–Whitney test to compare non-parametric continuous data and χ^2^ test to compare categorical data. We conducted simple and multiple regression analyses to investigate the association between reporting of funding by papers and the journal’s characteristics (except for the variable ‘requirement of disclosure of conflicts of interest’ due its high correlation with the variable ‘requirement of disclosure of source of funding’).

## Results

### Survey of HPSR papers

Table 1 shows the general characteristics of the 400 included HPSR papers. The median number of authors per paper was 4 (interquartile range 3–6). Systematic reviews had a significantly higher median number of authors compared with primary studies (*p* = 0.048). The majority of papers’ first authors was affiliated with public academic institutions (*n* = 288, 72%), with that percentage being lower in primary studies compared with systematic reviews (68% vs. 77%). A minority of papers’ first authors was affiliated with private for-profit (*n* = 5, 1%) and intergovernmental organisations (*n* = 1, 0.3%). The majority of first authors were from high-income countries (*n* = 368, 92%).

**Table 1:** General characteristics of the 400 included studies

	All *n* = 400	Primary studies *n* = 200	Systematic reviews *n* = 200	*P* value^b^
Number of authors, median (interquartile range)	4 (3–6)	4 (3–6)	5 (3–7)	0.048
	*n* (%)	*n* (%)	*n* (%)	
First author affiliations^a^				
Private academic institution	71 (18)	46 (23)	25 (13)	0.006
Public academic institution	288 (72)	135 (68)	153 (77)	0.045
Government	40 (10)	18 (9)	22 (11)	0.505
Not-for-profit organisation	33 (8)	23 (12)	10 (5)	0.018
Private for profit	5 (1)	2 (1)	3 (2)	0.999
Intergovernmental	1 (0.3)	1 (1)	0 (0)	0.999
Classification of country of affiliation of the first author				0.909
High income	368 (92)	183 (91)	185 (92)	
Upper-middle income	18 (5)	10 (5)	8 (4)	
Lower-middle income	9 (2)	4 (2)	5 (3)	
Low income	5 (1)	3 (2)	2 (1)	

^a^ More than one option could apply

^b^Comparison between primary studies and systematic reviews

**Fig. 1:**
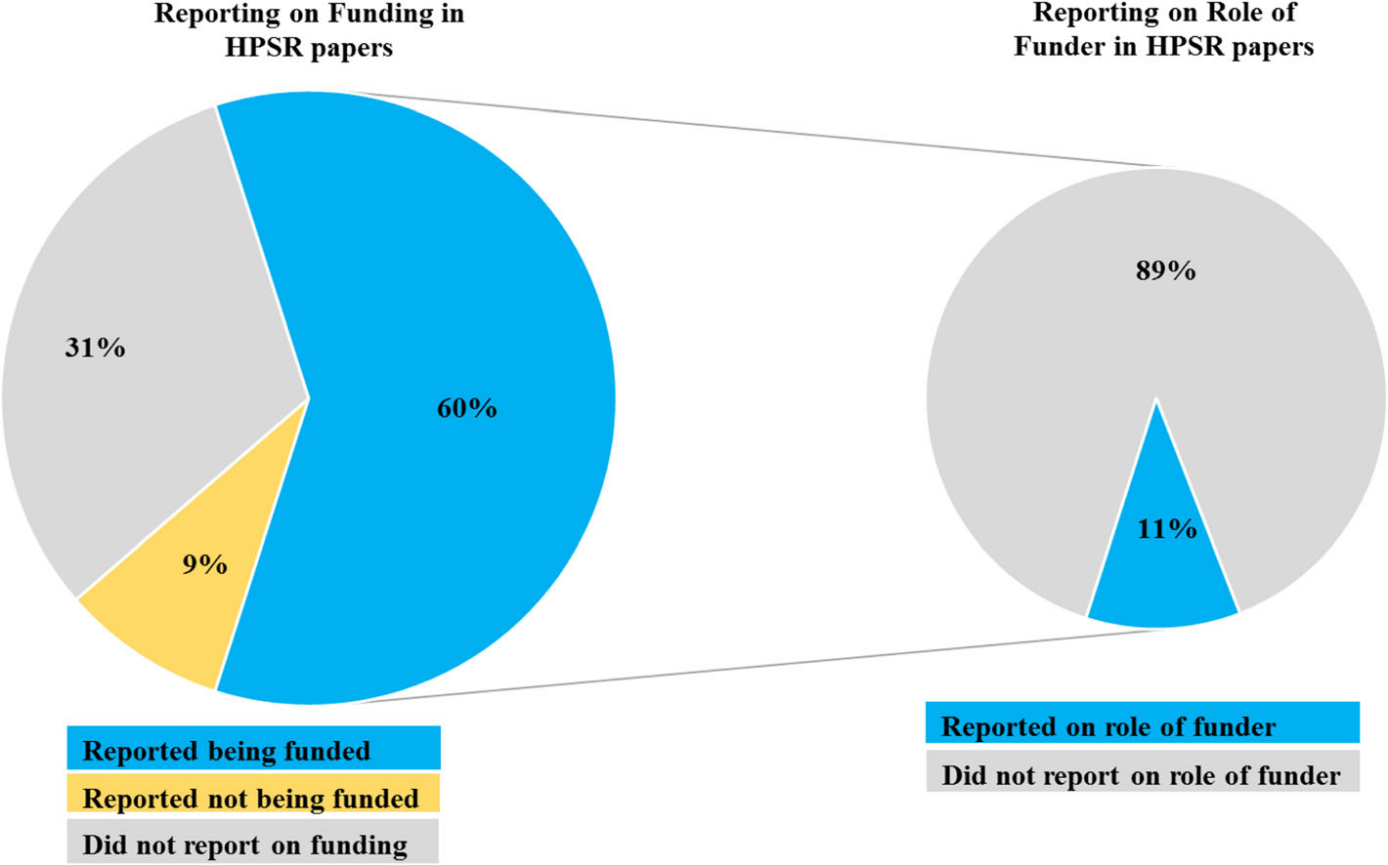
Reporting of funding source and role of funders in the 400 health policy and systems research (HPSR) papers

Table 2 presents the reporting of funding in the 400 included HPSR papers. A substantive percentage of papers (*n* = 126, 31%) did not report any detail on the study funding. As for papers that reported being funded (*n* = 240, 60%), their percentage was higher among primary studies compared to systematic reviews (65% vs. 54%). Among the 240 funded papers, the majority were funded by government (*n* = 186, 78%). Most of the studies did not report on the role of funders (*n* = 214, 89%) (Fig. 1).

**Table 2:** Reporting of funding in the 400 included studies

	All	Primary studies	Systematic reviews	*P* value^b^
	*n* (%)	*n* (%)	*n* (%)	
Study funding		*n* = 200	*n* = 200	0.001
Not reported	126 (31)	62 (31)	64 (32)	
Reported as funded	240 (60)	131 (65)	109 (54)	
Reported as not funded	34 (9)	7 (4)	27 (14)	
Source of funding^a^	*n* = 240	*n* = 131	*n* = 109	
Internal fund	42 (18)	21 (16)	21 (19)	0.511
Governmental	186 (78)	101 (77)	85 (78)	0.871
Private for-profit	10 (3)	4 (3)	6 (6)	0.345
Private not-for-profit	71 (30)	37 (28)	34 (31)	0.618
Role of funder	*n* = 240	*n* = 131	*n* = 109	
Not reported	214 (89)	121 (93)	93 (85)	0.061
Reported as a general statement of ‘no involvement’	4 (2)	3 (2)	1 (1)	
Reported as a specific statement of involvement in any of the stage(s) of the research	22 (9)	7 (5)	15 (14)	

^a^ More than one option could apply

^b^Comparison between primary studies and systematic reviews

Table 3 presents the status of reporting of funder involvement in each of the 15 stages of the research in studies that reported on involvement (whether positive or negative) in at least 1 of the stages. There was no reporting of the status of involvement (positive or negative) for 6 of the 15 stages of research.

**Table 3:** Status of the reporting of funder involvement in each of the 15 stages of the research, among studies that reported on any such involvement (whether positive or negative) (*n* = 22)

Stage of the research process	Not involved	Involved
1. Protocol/design of the study	19 (86)	0
2. Data collection	15 (68)	0
3. Data analysis/interpretation/management	19 (86)	0
4. Funded a writer	0 (0)	0
5. Preparation of the manuscript	20 (91)	0
6. Review of the manuscript	2 (9)	1 (5)
7. Approval of the manuscript	2 (9)	0
8. Decision to submit the manuscript	17 (77)	1 (5)
9. Verified data accuracy/fact checking	0	0
10. Auditing of study conduct	0	0
11. Conduct of study	0	0
12. Study oversight	0	0
13. Logistical support	0	0
14. Team assembly	0	0
15. Management	1 (5)	1 (5)
16. Any of the above	20 (91)	2 (9)
Median number of research stages reported per study	5 (4–5)	

### Survey of journals publishing HPSR papers

**Fig. 2:**
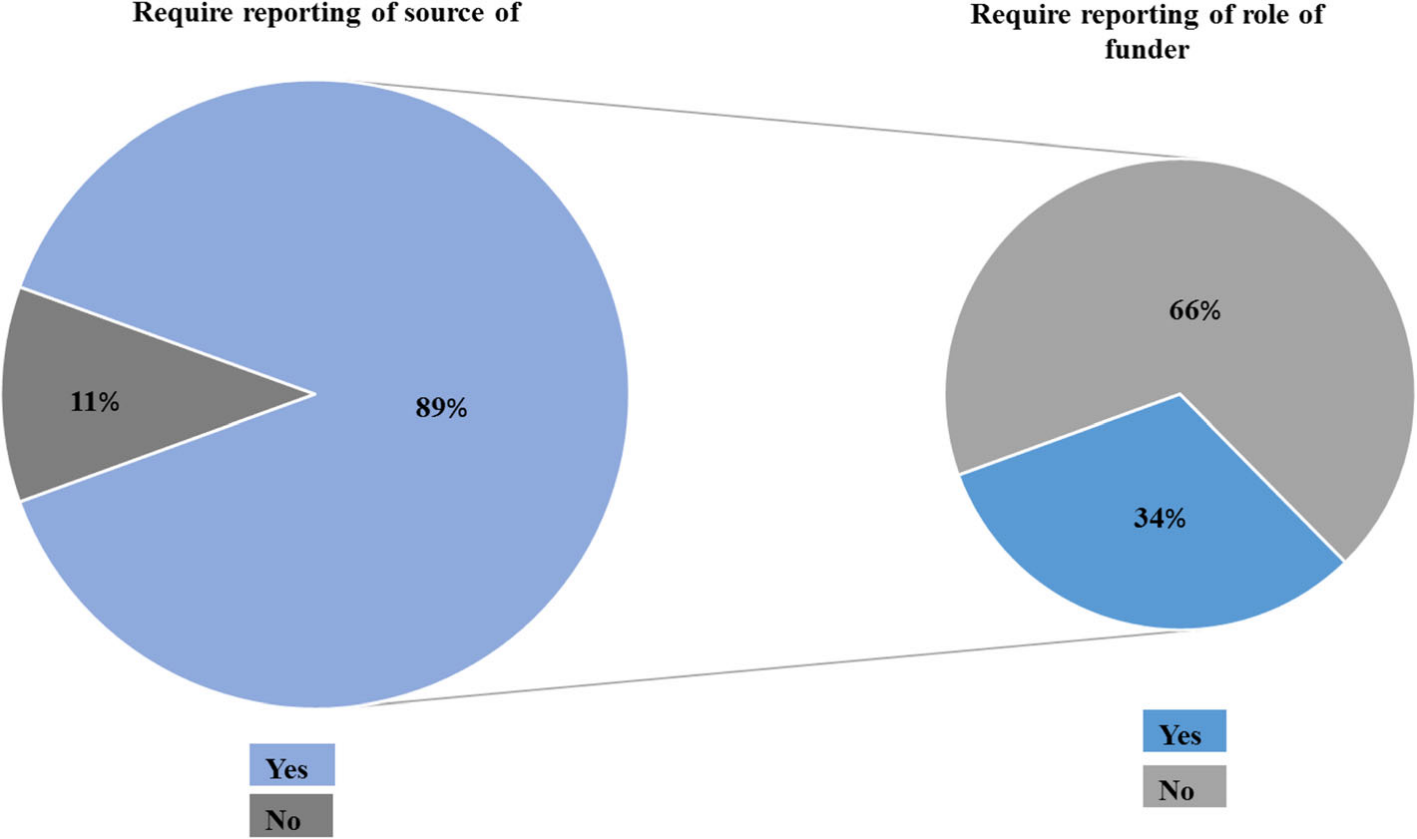
Requirements for the reporting of funding source and role of funders by the 198 journals publishing on health policy and systems research (HPSR)

Table 4 shows the characteristics of journals that published the 400 included HPSR studies (*n* = 198). It also includes results for the 2 overlapping subgroups of journals that published the 200 primary studies (*n* = 55) and the 200 systematic reviews (*n* = 153), respectively. Out of 198 journals publishing on HPSR, the majority required the reporting of source of funding (*n* = 176, 89%). Of those that did, 34% (56 out of 176) required the reporting of the role of funders (Fig. 2). That percentage was 19% (10 out of 53) for journals that published the primary studies and 37% (49 out of 133) for journals that published the systematic reviews.

**Table 4:** The characteristics of journals that published articles on health policy and systems research (HPSR) studies

	Journals of all 400 studies	Journals of the 200 primary studies	Journals of the 200 systematic reviews
Unique articles journals	198	55	153
Journal is an HPSR journal	55 (28)	55 (100)	11 (7)
Journal impact factor	1.90 (1.30–2.82)	2.15 (1.48–2.68)	1.92 (1.23–3.05)
Membership of ICMJE	40 (20)	4 (7)	37 (24)
Membership of COPE	154 (78)	44 (80)	119 (78)
Affiliation with a professional organisation	132 (67)	33 (60)	106 (69)
Journal requires disclosure of conflicts of interest	189 (96)	53 (96)	146 (95)
Journal requires reporting of source of funding	176 (89)	53 (96)	133 (87)
Journal requires reporting of role of funder	56 (34)	10 (19)	49 (37)

*COPE* Committee on Publication Ethics, *ICMJE* International Committee of Medical Journals Editors

Table 5 compares the characteristics of HPS journals (according to the Web of Science) (*n* = 72) and non-HPS journals that published the HPSR studies included in our first survey (*n* = 142). Only 15% of the HPS journals required reporting of the role of funders, compared to 32% of the non-HPS journals that published the HPSR studies.

**Table 5:** Comparison between the characteristics of journals listed as Health Policy and Services (HPS) and non-HPS journals that published health policy and systems research (HPSR) studies

	HPS journals	Non-HPS journals that published HPSR studies^a^	*P* value
Number of journals	72	142	
Journal impact factor	1.62 (1.02–2.32)	1.92 (1.26–3.05)	0.062
Membership of ICMJE	5 (7)	36 (25)	0.001
Membership of COPE	54 (75)	109 (77)	0.775
Affiliation with a professional organisation	45 (63)	98 (69)	0.339
Requires disclosure of conflicts of interest	67 (93)	135 (95)	0.545
Requires reporting of source of funding	65 (90)	122 (86)	0.364
Requires reporting of role of funder	11 (15)	46 (32)	0.007

^a^Journals that published HPSR papers but are not classified as HPS journals by the Web of Science

*COPE* Committee on Publication Ethics, *ICMJE* International Committee of Medical Journals Editors

### Regression analysis

Table 6 presents the simple and multiple logistic regression of ‘reporting of funding’ in included studies with the journals’ funding policies and other covariates. ‘Reporting of funding’ was significantly associated with publication in journals with higher impact in the simple logistic regression (odds ratio (OR) 1.15, 95% confidence interval (CI) 1.00–1.33) but not in the multiple logistic regression (OR 1.13, 95% CI 0.97–1.31).

**Table 6:** Simple and multiple logistic regression of ‘reporting of funding’ in included studies with journals’ funding policies and other covariates

Reporting of funding	Unadjusted OR (95% CI)	Adjusted OR (95% CI)
Systematic review versus primary study ^a^	0.95 (0.63–1.46)	0.95 (0.61–1.48)
Journal impact factor	1.15 (1.00–1.33)	1.13 (0.97–1.31)
Membership of ICMJE	1.15 (0.63–2.08)	1.08 (0.58–2.01)
Membership of COPE	1.09 (0.65–1.81)	1.15 (0.66–1.98)
Affiliation with a professional organisation	1.12 (0.72–1.74)	1.03 (0.65–1.63)
Journal requires disclosure of conflicts of interest	1.73 (0.63–4.76)	^b^
Journal requires reporting of source of funding	2.04 (0.99–4.24)	1.73 (0.80–3.77)
Journal requires reporting of role of funder	1.28 (0.77–2.12)	1.12 (0.66–1.92)

^a^Primary study is the reference category

^b^Variable not included in the model due to its high correlation with ‘Journal requires reporting of source of funding’

*CI* confidence interval, *COPE* Committee on Publication Ethics, *ICMJE* International Committee of Medical Journals Editors, *OR* odds ratio

## Discussion

### Summary of findings

Approximately one-third of HPSR papers did not report any detail on study funding. Of those that did, only 11% reported on the role of funders. Although the vast majority of journals publishing on HPSR required reporting of the source of funding, only approximately one-third of these journals required reporting of the role of funders. Journals classified under the HPS category were less likely than non-HPS journals that published the HPSR studies to require the reporting of the role of funders. We did not find any of the journal’s characteristics to be associated with the reporting of funding by papers.

### Strengths and limitations

This is the first study to address the reporting of funding in HPSR papers. Additionally, it is the first to assess the policies on reporting of funding of journals publishing on HPSR. We used duplicate and independent selection and data abstraction processes to ensure the validity of findings. In addition, conducting the two surveys in parallel enabled us to assess the association between the reporting of funding and the funding policies of journals. One possible limitation of this study is that journals might inform the authors about certain requirements on reporting of funding at the time of submission or acceptance that might not be reflected in the policies published on their website.

### Interpretation of findings

While a third of HPSR papers did not provide any information about funding, most journals in which they were published had adequate policies requiring the reporting of the funding source. This reflects both a suboptimal compliance by authors with the funding policies and a deficient implementation by the journals. On the other hand, the low reporting of the role of funders (11%) seems to be related to the inadequacy of the journals’ policies since approximately one-third of the journals required the reporting of the role of funders. Journals classified under the HPS category were less likely than the non-HPS journals that published the HPSR studies to require reporting of the role of funders. This highlights the need for health policy journals to strengthen their policies for the reporting of research funding.

### Comparison to other studies

HPSR papers do not compare favourably with clinical papers in terms of the reporting of funding. Recently, we found that 89% of clinical trial reports published in 2015 included funding statements [16], a percentage substantively higher than the 69% found for HPSR papers herein. In addition, we found that 50% of the clinical trials described as funded reported on the role of funders, a much higher percentage than the 11% found for HPSR papers herein [16]. Another study found that 35% of funded surgical trials published in 10 surgery journals reported on the role of study sponsors [9].

Most HPS journals have policies that require the reporting of source of funding (90%). While we did not identify any similar publication for clinical journals, one study found that 90% of public health journals have policies for reporting study funding [17]. On other hand, the percentage of journals requiring the reporting of the role of funders was low for both HPS (15%) and public health (23%) [17].

Reporting of funding’ was significantly associated with publication in journals with higher impact factor in the simple but not in the multiple logistic regression. Similarly, we previously found that the core clinical journals had better reporting of funding associated with the impact factor of the journal (OR 1.41, 95% CI 1.09–1.9) [16]. One potential explanation of this finding is that journals with high impact factors are more likely to enforce the implementation of policies related to the declaration of funding.

## Conclusions

### Implication for practice

Our study identified some gaps in the funding policies of HPS journals that are associated with low reporting of funding information in HPSR papers. HPS journals need to implement more detailed policies requiring authors to report relevant details such as the role of funders, type of support (e.g. monetary, provision of supply) and amount of fund [16]. Moreover, journals need to better enforce their funding policies. We have previously proposed the type of funding information that should be reported, along with a fillable PDF document as a standardised instrument for the reporting of funding information. Additionally, reporting statements (such as PRISMA and CONSORT) could require more details, e.g. in terms of the specific roles that the funder plays.

### Implications for future research

Future research could assess the reasons for low reporting of funding information in HPSR, as well as the accuracy and completeness of the reported funding information. Moreover, it would be interesting to assess the effectiveness of interventions to improve reporting of funding such as the use of standardised reporting instruments.

## Abbreviations

CI: confidence interval
HPS: health policy and services
HPSR: health policy and systems research
OR: odds ratio
